# Comparative diagnostic accuracy study of point of care ultrasound techniques for detection of left atrial enlargement by hospital medicine physicians from archived echocardiogram images

**DOI:** 10.1002/jhm.70245

**Published:** 2025-12-13

**Authors:** Christopher J. Smith, Jesse Umbra, Sofia Quintero, Austin Wilson, Elizabeth Lyden, Nidish Tiwari, Brian Shahan, Jana Wardian

**Affiliations:** ^1^ Department of Internal Medicine, Division of Hospital Medicine University of Nebraska Medical Center Omaha Nebraska USA; ^2^ College of Allied Health Professions, Diagnostic Medical Sonography University of Nebraska Medical Center Omaha Nebraska USA; ^3^ Department of Internal Medicine, Internal Medicine Residency Program University of Nebraska Medical Center Omaha Nebraska USA; ^4^ College of Public Health, Department of Biostatistics University of Nebraska Medical Center Omaha Nebraska USA; ^5^ Department of Internal Medicine, Division of Cardiology University of Nebraska Medical Center Omaha Nebraska USA

## Abstract

**Background:**

Left atrial enlargement (LAE) is predictive of cardiovascular morbidity and mortality. Prior studies of point‐of‐care ultrasound (POCUS) interpretation methods for identifying LAE utilized older echocardiographic reference ranges.

**Objectives:**

Compare the test characteristics of hospitalist‐performed POCUS techniques for identifying LAE as compared to contemporary echocardiographic reference ranges.

**Methods:**

Fully paired, comparative diagnostic accuracy study of two index tests applied to archived echocardiogram images: visual assessment of the left atrium to aorta diameter (LAE sign) and left atrial (LA) anteroposterior diameter >4 cm in the parasternal long axis view. The reference test was moderate to severe LAE by echocardiography‐derived left atrial volumetric index.

**Results:**

After exclusion criteria, 239 of 321 (74.5%) exams were included. The LAE sign had a sensitivity, specificity, positive predictive value (PPV), and negative predictive value (NPV) of 67.5%, 71.4%, 32.1%, and 91.6%. LA diameter of >4 cm had a sensitivity, specificity, PPV, and NPV of 87.5%, 75.9%, 42.2%, and 96.8%. The difference in sensitivity (*p* = .005) and specificity (*p* = .049) between the index tests was statistically significant. The diameter measurement had better positive and negative likelihood ratios (LR + 3.63, LR−0.16) than the LAE sign (LR + 2.36, LR− 0.46).

**Conclusions:**

Both POCUS techniques for diagnosing LAE performed reasonably well compared to current echocardiographic reference ranges, with LA diameter >4 cm having better sensitivity and specificity than visual estimation of the LAE sign. These tests can help identify patients at risk for cardiovascular disease who may benefit from echocardiogram referral.

## INTRODUCTION

Detection of left atrial enlargement (LAE) is a common application of cardiac point‐of‐care ultrasound (POCUS).^1^, ^2^ LAE is not a part of the normal aging process, but rather the result of pathological pressure and volume overload leading to remodeling of the atrium.^3^ Studies have reported the incidence of new‐onset LAE in a general population is 11.8% over 10 years^4^ and the pooled prevalence in patients with hypertension is 32%.^5^ LAE has been referred to as the “hemoglobin A1c of the heart” because it is a marker of poor cardiovascular health. The presence of LAE predicts various adverse outcomes, including atrial fibrillation, stroke, heart failure, hypertensive heart disease, and cardiovascular mortality across various cohorts of patients.^3^, ^6^, ^7^

Because of its importance in predicting cardiovascular pathology and adverse outcomes, there is great interest in identifying LAE by noncardiologists using POCUS. The echocardiographic gold standard for determining LAE is calculating the left atrial volumetric index (LAVI) using the biplane disk summation technique, but this application is beyond the scope of most POCUS operators. Importantly, the LAVI ranges defining LAE have changed in recent years, reflecting observations that LA volumes in a normal population are higher than previously recognized.^8^, ^9^

One of the most widely described POCUS techniques for identifying LAE is via visual estimation of the left atrium to aorta diameter (LAAD), referred to as the LAE sign. Using this approach, LAE is present if the LA anterior‐posterior (AP) depth is larger than the overlying aortic root throughout the cardiac cycle in the parasternal long axis view (PLAX) (i.e., LAAD > 1 in end‐diastole). ^10^ The LAE sign can help in the diagnosis of many commonly encountered cardiopulmonary disorders.^11^ A study by Han in 2019 found that the presence of the LAE sign was associated with increased 5‐year mortality and could be used as a screening method for echocardiography referral.^12^ Another study found that a multi‐system “quick look” POCUS exam including the LAE sign was related to complex hospitalization.^13^

While assessing for the LAE sign is appealing for POCUS operators because it is intuitive and quick, reported test characteristics are modest with sensitivity (Sn) and specificity (Sp) of approximately 60%–80%. ^10^, ^14^ It should also be noted that these studies used older LAVI reference ranges with lower thresholds for LAE than contemporary guidelines.^9^ Furthermore, LAE interpretation in these studies was conducted by cardiologists, which may not reflect the performance of non‐cardiologist POCUS operators.

Historically, linear measurement of the LA AP diameter in the PLAX view was used to assess LAE with >4 cm considered abnormal. Since the LA does not necessarily enlarge symmetrically, this measurement can underestimate LA volume,^15^ and is therefore no longer recommended in echocardiography guidelines.^8^, ^9^ However, this linear measurement remains an appealing option for noncardiologist POCUS‐users ^16^ because of its simplicity and reproducibility, and it is still used in certain clinical contexts^17^, ^18^, ^19^ and retrospective studies.^20^

Identification of LAE by POCUS has clinical value in identifying patients at risk for poor outcomes who may benefit from further cardiovascular evaluation; however, the ability of POCUS techniques to identify LAE as compared to current echocardiographic classifications is unclear. Therefore, the objective of this study was to determine and compare the test characteristics of two index tests performed by non‐cardiologist POCUS‐users: (1) visual assessment of the LAE sign and (2) semiquantitative measurement of the LA diameter >4 cm for identifying moderate to severe LAE in a population of patients referred for transthoracic echocardiogram (TTE) as compared to contemporary echocardiography‐derived LAVI reference ranges.

## MATERIALS AND METHODS

### Study design and participants

We conducted a within‐subject (fully paired), comparative diagnostic accuracy study of the index tests, as described below. Index tests were prospectively performed on images from archived TTEs. Eligibility criteria for inclusion were consecutive, complete, adult TTEs performed at a 700‐bed tertiary care hospital starting on January 1, 2022. Both inpatient and outpatient TTE referrals were included. TTEs were performed by registered diagnostic cardiac sonographers and originally interpreted by board‐certified cardiologists. Deidentified images, patient characteristics, and TTE report findings were exported from the health system's echocardiography management system, IntelliSpace Cardiovascular (Philips Healthcare), for review. Exclusion criteria included duplicate exams from the same patient, studies with missing LAVI data, altered cardiac anatomy (heart transplant, mechanical valve replacements, left ventricular assist devices, and intra‐atrial devices), and uninterpretable images (based on a pre‐enrollment review of images by an advanced cardiac sonographer). The study was approved by the institution's IRB.

### Test methods

**Figure 1:**
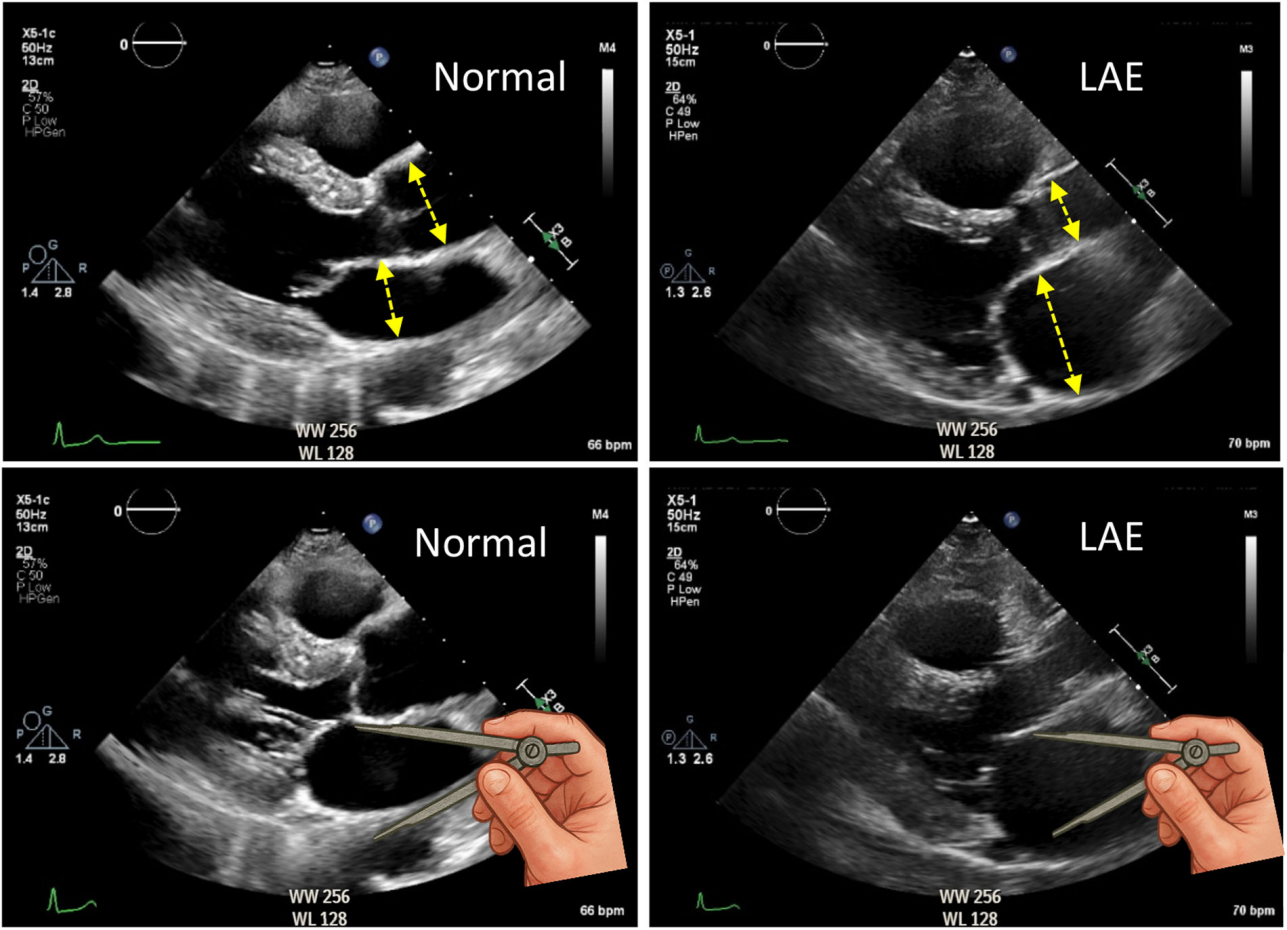
Top figures illustrate visual estimation of LAE sign in the parasternal long axis view. A left atrium to aorta diameter ratio >1 throughout the cardiac cycle was considered a positive test. Bottom figures illustrate measuring left atrium diameter in the parasternal long axis view. A caliper was calibrated to a 4 cm reference using the depth markers on the side of the image. The caliper distance was then compared to the diameter of the left atrium at end‐ventricular systole. LAE, Left atrial enlargement.

TTE images from the PLAX view were prospectively reviewed by one of three blinded hospital medicine physicians with hospital privileges in cardiac POCUS. Reviewers first visually estimated the LAAD for the LAE sign. A LAAD > 1 throughout the cardiac cycle was considered a positive test for LAE (Figure 1).

Next, the reviewer performed a semiquantitative linear measurement of the LA AP diameter in end‐ventricular systole. Electrocardiogram calipers were calibrated to 4 cm using the depth scale markers on the side of the echocardiogram image. The caliper was compared to the AP diameter of the LA at end‐ventricular systole, perpendicular to the aortic root long‐axis at the level of the aortic sinuses^9^ (Figure 1). A LA diameter >4 cm was considered positive for LAE, consistent with previously recommended cut points.^21^

The reviewers' findings were compared to the presence of moderate to severe LAE from the TTE report, defined as LAVI > 41 mL/m^2^ using the biplane disk summation technique.^8^, ^9^ We used moderate to severe LAE as our reference standard because there is overlap between normal and pathologic LA remodeling. This is reflected in the British Society of Echocardiography guidelines that define a “borderline” LAVI of 34–38 mL/m^2^ which would fall into the “mildly abnormal” category of the American Society of Echocardiography ranges.^8^ To calculate inter‐rater reliability (IRR), one‐third of eligible TTE exams were evaluated by a second blinded reviewer.

Secondary outcomes of interest were test characteristics for detection of any severity of LAE (LAVI > 34 mL/m^2^) and severe LAE (LAVI > 48 mL/m^2^). As part of post hoc analysis, a cardiac sonographer measured the linear LA AP diameter from a subset of TTE exams (*n* = 80) to compare test characteristics to the semi‐quantitative caliper method. We also analyzed the subset of cases with aortic root dilation >4 cm and those with normal TTE reports, defined as left ventricular (LV) ejection fraction >50%, normal diastolic function, no moderate or severe mitral or aortic stenosis, no moderate to severe mitral or aortic regurgitation, no MV prolapse, and no LV hypertrophy (LV mass index <96 g/m^2^ female or <116 g/m^2^ men) to determine basic counts and cross‐reference with the index tests.

Reviewers participated in a 1‐h training session of LAE assessment techniques before beginning the study. Online training materials remained available as a reference. Reviewers used the same video player (VideoLan, VLC Media Player, 3.0.21) for consistency. Reviewers were blinded to patient data and TTE report findings, including LAVI calculations.

### Analysis and reporting

We performed a power analysis based on 13,046 TTEs performed at our institution in 2022; the prevalence of moderate to severe LAE was 15%. A sample size of 240 achieved 83% power to detect an increase in Sn from 0.6 to 0.8 using a one‐sided binomial test. Additionally, it achieved 87% power to detect an increase in Sp from 0.5 to 0.6 using a one‐sided binomial test with a target significance level of 0.05. We estimated that approximately 25% of cases would meet exclusion criteria, resulting in a final sample size of *n* = 320.

Descriptive statistics were used to summarize patient characteristics and cardiac findings from the TTE report. Estimates of Sn, Sp, positive predictive value (PPV), negative predictive value (NPV), positive likelihood ratio (LR+) and negative likelihood ratio (LR−) were reported with 95% confidence intervals. The kappa statistic was used to evaluate the diagnostic agreement between the index tests and reference test. Sn and Sp values of the index tests were compared using McNemar's test. The kappa statistic was used to assess IRR for the index tests. All analysis was done using SAS Version 9.4. A *p*‐value < .05 was considered statistically significant. The Standards for Reporting of Diagnostic Accuracy Studies guidelines,^22^ a quality assessment tool^23^ and a literature review^24^ for comparative diagnostic accuracy studies were used as framework for reporting study results.

## RESULTS

A total of 321 TTE exams were reviewed with 82 (25.5%) meeting exclusion criteria, resulting in 239 exams included in the analysis. Exams were excluded for the following reasons: missing LAVI data, *n* = 38; altered cardiac anatomy, *n* = 35; uninterpretable image, *n* = 29; missing images, *n* = 2 (note that some cases met more than one exclusion criteria). TTE findings of study exams are displayed in Table 1. Approximately 17% of exams had moderate to severe LAE by LAVI measurement. Table 2 presents the joint classification (also known as a two‐by‐four table)^24^, ^25^ of the two index tests categorized by the presence or absence of moderate to severe LAE on the reference test.

**Table 1:** Patient characteristics and transthoracic echocardiography findings (*n* = 239).

Characteristic	Value
Female, *n* (%)	115 (48.1)
Age mean, years (IQR)	59.3 (22)
BMI mean, kg/m^2^ (SD)	30.5 (8.1)
Left atrial volume index, *n* (%)	
≤34 mL/m^2^ (normal)	167 (69.9)
34–41 mL/m^2^ (mild enlargement)	32 (13.4)
41–48 mL/m^2^ (moderate enlargement)	20 (8.4)
>48 mL/m^2^ (severe enlargement)	20 (8.4)
Diastolic function, *n* (%)	
Normal	91 (38.1)
Grade 1 dysfunction	54 (22.6)
Grade 2 dysfunction	28 (11.7)
Grade 3 dysfunction	9 (3.8)
Not reported	57 (23.8)
Left ventricular ejection fraction, *n* (%)	
>50% (normal)	180 (75.3)
40%–50% (mild dysfunction)	23 (9.6)
30%–39% (moderate dysfunction)	19 (7.9)
<30% (severe dysfunction)	14 (5.9)
Not reported	3 (1.3)

**Table 2:** Joint classification for identifying moderate to severe LAE.

	LAE present (Reference)			LAE absent (Reference)		
	Diameter+	Diameter−	Total	Diameter+	Diameter−	Total
LAE sign+	27	0	27	42	15	57
LAE sign−	8	5	13	6	136	142
Total	35	5	40	48	151	199

*Note*: Left atrial enlargement (LAE), diameter = anteroposterior diameter of >4 cm by caliper measurement. Reference standard was presence of moderate to severe LAE as determined by echocardiography.

Primary outcomes: The test characteristics for the index tests are listed in Table 3. Visual estimation of the LAE sign had a Sn, Sp, PPV, and NPV of 67.5%, 71.4%, 32.1%, and 91.6%. An LA AP diameter of >4 cm by caliper measurement had a Sn, Sp, PPV, and NPV of 87.5%, 75.9%, 42.2%, and 96.8%. The difference in Sn and Sp between the 2 index test methods was statistically significant (Sn 67.5% vs. 87.5%, *p* = .005; Sp 71.4% vs. 75.9%, *p* = .049). The caliper measurement had better positive and negative likelihood ratios (LR+ 3.63, LR− 0.16) than the LAE sign (LR+ 2.36, LR− 0.46). The LAE sign had fair agreement (*κ* = 27.0, confidence interval [CI]: 15.0–39.0) with TTE findings, whereas the linear measurement had moderate agreement (*κ* = 44.3, CI: 32.8–55.9). Both tests had substantial^26^ IRR with kappa coefficients of about 63%. The LAE sign and caliper measurement had substantial agreement with one another (*κ* = 0.73).

**Table 3:** Index test characteristics for identifying moderate to severe LAE.

	Sens, %	Spec, %	LR+	LR−	PPV, %	NPV, %	Agreement^a^	IRR^a^
	(95% CI)	(95% CI)	(95% CI)	(95% CI)	(95% CI)	(95% CI)	(95% CI)	(95% CI)
LAE Sign	67.5* (53.0–82.0)	71.4* (65.1–77.6)	2.36 (1.73–3.2)	0.46 (0.29–0.72)	32.1 (22.2–42.1)	91.6 (87.3–96.0)	27. 0 (15.0–39.0)	62.8 (44.6–80.9)
Diameter >4 cm	87.5* (77.3–97.8)	75.9* (69.9–81.8)	3.63 (2.76–4.77)	0.16 (0.07–0.38)	42.2 (31.5–52.8)	96.8 (94.0–99.6)	44.3 (32.8–55.9)	62.9 (44.0–81.8)

Abbreviations: IRR, interrater reliability; LAE, left atrial enlargement; LR+, positive likelihood ratio; LR−, negative likelihood ratio; NPV, negative predictive value; PPV, positive predictive value.

^a^ Kappa statistic.

* *p*‐value < .05.

Secondary outcomes: For detecting any severity LAE (LAVI > 34 mL/m^2^), the test characteristic for the LAE sign and caliper measurement were Sn 62.5%, Sp 76.7%, PPV 53.6%, NPV 82.6% and Sn 70.8%, Sp 80.8%, PPV 61.5%, and NPV 86.5%, respectively. For detecting severe LAE (LAVI > 48 mL/m^2^), the LAE sign had Sn 75%, Sp 68.5%, PPV 17.9%, NPV 96.8% and the caliper measurement had Sn 90%, Sp 70.3%, PPV 21.7%, NPV 98.7%.

In post hoc analysis of the subset of cases with sonographer‐derived linear measurement of LA AP diameter >4 cm, the test characteristics for detecting moderate to severe LAE were similar to the caliper method with Sn 87.5%, Sp 70.3%, PPV 42.4%, and NPV 95.7%. The level of agreement between the linear measurement and reference test was moderate (*κ* = 0.41). The level of agreement between the directly measured diameter >4 cm and caliper measurements >4 cm was moderate (*κ* = 0.52).

The number of study cases with normal TTE findings was 52/239 (21.8%). Of these, 39/52 (75%) had neither LAE sign, nor caliper measurement >4 cm. The LAE sign was present in 10/52 (19.2%) and the caliper measurement >4 cm was present in 13/52 (25%), with 10 cases having both LAE sign and caliper findings. Of these cases, 3/10 (30%) with LAE sign and 4/13 (30.8%) with caliper >4 cm had moderate to severe LAE by TTE‐derived LAVI measurement.

There were 11/228 (4.8%) cases with aortic root dilation >4 cm (range 4.0–4.5 cm) reported on the TTE report, of which five cases had moderate to severe LAE by LAVI measurement. The LAE sign correctly identified 3/5 (60%) and caliper measurement correctly identified 4/5 (80%) of these cases. There were no false positive cases for either index test when aortic root dilation was present.

## DISCUSSION

This study found that two commonly used POCUS interpretation methods performed reasonably well in identifying moderate to severe LAE as compared to echocardiographic assessment. These findings are in line with previously reported test characteristics for visual assessment of LAE sign^10^ and hospitalist‐performed linear measurements of the LA diameter^27^ for detection of moderate to severe LAE, as well as the known limitations of extrapolating LA diameter to a volumetric measurement.^15^ While test characteristics for both strategies were fair, the caliper measurement of AP diameter outperformed visual estimation of the LAE sign, including improved Sn and Sp. When determining the clinical usefulness of a diagnostic test, likelihood ratios are particularly relevant, as they integrate both the Sn and Sp into their calculation, and the result can be used to determine a post test probability of a diagnosis.^28^ With its LR− approaching 0.1, a LA diameter of <4 cm substantially lowers the posttest probability of moderate to severe LAE.^29^ For example, if a clinician evaluates a previously healthy 50‐year‐old male for dyspnea and estimates the pre‐test probability of LAE as a marker for cardiac pathology is 20%, a normal diameter measurement would lower the post‐test probability to 3.8%, thereby supporting a decision to forego TTE referral. The LR+ for both index tests was between 2 and 5, which generates modest, but potentially meaningful changes in post‐test probability.^29^ Using the same pretest probably of 20%, a positive LAE sign results in a posttest probability of 37.1% and diameter >4 cm more than doubles the posttest probability to 47.6%, which may compel a clinician to pursue additional testing.

Given its prevalence, lack of reliable physical exam findings, and association with various adverse cardiovascular outcomes, evaluation for LAE should be included as part of the systematic interpretation of cardiac ultrasound examinations. Recognition of moderate to severe LAE can improve the care of hospitalized patients by expediting discovery of cardiac pathology (e.g., left ventricular diastolic dysfunction, valvular disease), as well as serving as a prognostic marker for serious cardiovascular outcomes (e.g., atrial fibrillation, stroke, and mortality)^3^, ^12^ and complex hospitalization.^13^ Detection of LAE may therefore lead to changes in counseling, medications, and monitoring in both the inpatient and outpatient settings to more aggressively address the root cause of LAE.

When integrating these findings into clinical practice, we advocate that capable POCUS operators utilize measurement of the LA AP diameter, as it has superior test characteristic compared to visual assessment of the LAE Sign. In situations where the diameter cannot be routinely measured (e.g., time constraints, novice operator), a practical approach could be starting with visual estimation of the LAE sign and subsequently measuring the AP diameter for borderline or uncertain cases. Based on the results, patients with a high posttest probability for new LAE should be referred for TTE to confirm the diagnosis and evaluate for other abnormalities.^12^

As with other POCUS applications, there may be concern that assessment for LAE could lead to unnecessary subsequent testing and healthcare expenditures. In the current study, approximately 90% of exams with either LAE sign or a caliper dimeter >4 cm had abnormal findings on TTE. For context, approximately half of all TTE referrals at a large academic center had no significant abnormalities,^30^ suggesting the strategies described for detecting LAE are an effective way to identify patients in need of further evaluation. This is further supported by a prior study that demonstrated evaluation for the LAE sign can identify high‐risk patients and reduce the number of unnecessary referrals for TTE when combined with patient‐level variables (e.g., age, comorbid conditions).^12^ We also suggest that hospitalists compare cardiac ultrasound findings with prior TTE reports to ensure any abnormal findings are new before pursuing additional testing.

To our knowledge, this is the first study to directly compare commonly used POCUS interpretation methods using contemporary echocardiographic reference ranges. As noted previously, the American Society of Echocardiography chamber quantification guidelines published in 2015 lowered LAE severity ranges. For example, what was previously classified as “mild” LAE is now in the normal range, and what was considered “moderate” LAE would now be in the “mild” range. In the current study, the prevalence of LAE was roughly half of previous POCUS reports, resulting in lower PPV and higher NPV.^10^, ^14^

Our study also adds to the literature in that it assessed the performance of non‐cardiologists. Prior studies of the LAE sign have largely relied on cardiologists' evaluation, which may not reflect the experience of noncardiologist POCUS‐users. For example, a prior study found medical students performed worse than echocardiographers when interpreting cardiac POCUS exams for various pathologies, including LAE.^31^ While not surprising, this supports the need to consider the background of the reviewer when interpreting test characteristics.

There are important pros and cons to both index tests evaluated in this study. The primary advantage of the LAE sign is that it is quick and intuitive; however, there are potential challenges POCUS users should keep in mind. For one, it relies on comparing dynamic structures that are moving throughout the cardiac cycle, which can be especially challenging if tachycardia or arrhythmias are present. Additionally, it assumes the reference object (aorta) is normal size, although aortic root dilation did not seem to greatly impact the index test performance in the current study. Linear measurement of the AP diameter has the advantage of being more objective, although our semi‐quantitative caliper measurement resulted in a similar IRR as the LAE sign. Like the LAE sign, the linear LA AP diameter can also be effected by aortic root size.^32^ Additionally, the diameter measurement may require additional time and knowledge, however these skills should be attainable to many operators, as simple linear measurements are commonly used in a variety of POCUS applications.^33^, ^34^, ^35^ For both index tests, clinicians should be mindful of other situations that may lead to inaccurate or unreliable results. This includes operator (e.g., experience), technical (e.g., improper gain), and patient (e.g., obesity) characteristics that may impact image quality or interpretation.^6^

The current study had several limitations. It was conducted at a single site, which may limit generalizability. Similar to prior studies of the LAE sign,^10^, ^12^, ^14^, ^36^ echocardiographic images were acquired by registered diagnostic cardiac sonographers. While this means image quality may not be representative of POCUS‐users, it is unclear how this may impact the diagnostic performance of the index tests. We anticipate that POCUS operators are likely to have more exams with sub‐optimal image quality; however, it is uncertain if this would disproportionately impact patients with LAE. For example, obesity is a common cause of non‐diagnostic echocardiogram exams,^37^ and the percentage of patients in the current study with BMI > 30 was similar for those with (45%) and without (50%) moderate to severe LAE. In a prior study of hospitalist performing cardiac POCUS using handheld devices, only 5/322 (2%) of exams were indeterminate for moderate to severe LAE, suggesting POCUS operators can reliably attain images of adequate quality to make this determination.^27^ Future studies should examine this study's methods for detecting LAE using prospective images acquired by POCUS‐users at the bedside to validate our findings.

Another limitation was that the method for determining the LA diameter relied on semi‐quantitative caliper measurements. This may have led to imprecise categorization, especially for borderline cases near the 4 cm cut point.^36^ This approach was chosen primarily for practical reasons, as direct measurement of the LA diameter was not reliably reported as part of the echocardiography protocol at our institution, and we lacked the software to measure the absolute linear measurement from archived TTE exams. Despite this limitation, the IRR of this technique was substantial with a kappa value of 0.63. Other studies reporting IRR for different POCUS findings vary greatly based on the specific application and the background of the reviewers, but, as a comparison, the IRR value is similar to what has been reported for evaluation of the inferior vena cava^38^, ^39^ and pulmonary b‐lines ^40^ by emergency physicians. Furthermore, in a post‐hoc analysis the test characteristics of AP diameter >4 cm as measured by a cardiac sonographer had nearly identical test characteristics to the caliper method, supporting our findings are likely applicable to linear measurements. The moderate kappa agreement between the two tests likely reflects the small sample size and inter‐rater variance between sonographers and hospitalists.^41^ Future studies should explore the test characteristics of absolute linear measurements performed by the bedside clinician.

Additionally, the study reviewers were not blinded from their assessment of the individual index tests. Reviewers evaluated the LAE sign followed immediately by the caliper assessment, which may have introduced bias.^25^ Study designers attempted to mitigate this bias by (1) starting with the more subjective evaluation technique followed by the more objective technique and (2) instructing reviewers in the training session to avoid changing their results once recorded.

A final potential limitation was that we only evaluated images from the PLAX view. POCUS techniques for detecting LAE prioritize the PLAX view, in part because image acquisition tends to be easier than in other standard cardiac views. That said, parasternal views are not always attainable because of sternal incisions or dressings, lung disease, or chest wall deformities. Additionally, the PLAX view provides only a partial view of the LA leading to imprecision in evaluating for LAE. Apical views afford a more accurate representation of the LA dimensions, which is why they are used in LAVI calculations. Future studies should explore POCUS techniques for assessing LAE in other cardiac views.

In conclusion, we found that two POCUS methods performed reasonably well in diagnosing moderate to severe LAE, as compared to the current echocardiographic reference ranges. LA AP diameter >4 cm outperformed visual estimation of the LAE sign with statistically significant differences in Sn and Sp. We believe these tests can be used clinically to help identify patients at risk for cardiovascular disease who may benefit from echocardiogram referral.

Elements of this work have been presented at the following meetings in abstract form:

Society of Hospital Medicine Nebraska Chapter Abstract Competition. October 16, 2024. Omaha, NE.

Society of Hospital Medicine Converge 2025. April 23‐25, 2025. Las Vegas, NV.

## CONFLICT OF INTEREST STATEMENT

The authors declare no conflicts of interest.
